# Terminal ileum Burkitt's lymphoma related ileocolic intussusception in a five-year-old child: a case report and review of literature

**DOI:** 10.1016/j.ijscr.2025.112088

**Published:** 2025-10-21

**Authors:** Zemen Asmare Emiru, Amsalu Molla Getahun, Addisu Assfaw Ayen

**Affiliations:** a Department of anatomic pathology, Debre Tabor University, Ethiopia; b Department of general surgery, breast and endocrine surgery sub-specialist, Debre Tabor University, Ethiopia; c Department of internal medicine, Debre Tabor University, Ethiopia

**Keywords:** Burkitt lymphoma, Ileocolic intussusception, Intestinal polyp, Case report, Ethiopia

## Abstract

**Introduction and importance:**

Intussusception is a common pediatric condition characterized by the invagination of a proximal bowel segment into a distal segment, leading to intestinal obstruction and acute abdominal symptoms. While most cases are idiopathic, rare secondary causes, such as gastrointestinal Burkitt lymphoma, can occur.

**Presentation of case:**

A 5-year-old Ethiopian male presented in January 2024 with a 24-h history of crampy abdominal pain, vomiting, abdominal distension, and currant jelly stool. Examination revealed fever (38 °C), tachypnea (28 breaths/min), tachycardia (125 bpm), and abdominal tenderness. Ultrasound showed a target sign, and after resuscitation, the patient underwent surgery for suspected intussusception. Intraoperatively, ileocecal intussusception with an intraluminal mass serving as the lead point was identified and resected with primary anastomosis. Histopathology confirmed Burkitt lymphoma. Postoperative follow-up was uneventful.

**Clinical discussion:**

Burkitt lymphoma is a highly aggressive B-cell non-Hodgkin lymphoma that rarely presents as a lead point for ileocecal intussusception in children. While factors like immunocompromise and Epstein-Barr virus (EBV) are often implicated, sporadic cases, such as the one presented here, can occur without identifiable risk factors. Diagnosis typically relies on postoperative histopathology, and complete surgical resection is associated with excellent outcomes.

**Conclusion:**

Burkitt lymphoma is an aggressive B-cell non-Hodgkin lymphoma that can rarely cause secondary intussusception. Accurate postoperative evaluation and histopathological diagnosis, along with complete surgical removal followed by chemotherapy, are essential for a favorable outcome.

## Highlights

• This case highlights a rare presentation of primary gastrointestinal Burkitt lymphoma (GI-BL).
• Although rare, gastrointestinal Burkitt lymphoma can act as a secondary lead point for intussusception in pediatric patients.
• Intussusception with a GI-BL lead point poses diagnostic challenges, particularly preoperatively.
• Complete surgical resection of GI-BL can result in excellent outcomes with proper management.

## 1 Introduction

Intussusception is a condition in pediatric patients characterized by the invagination of a proximal segment of bowel into a distal segment, leading to intestinal obstruction and acute abdominal symptoms. The majority of intussusceptions are idiopathic; however, in 1.5–12.0 % of cases, a secondary pathological cause, identifiable as a lead point, is present [1]. Lymphoma is a rare secondary cause of intussusception, with only 10 % of non-Hodgkin lymphomas localized to the gastrointestinal tract [2]. In exceedingly rare instances, Burkitt lymphoma may manifest as an intestinal polyp, serving as the lead point and triggering secondary intussusception [3]. Gastrointestinal Burkitt lymphoma can manifest with a spectrum of symptoms ranging from nonspecific abdominal discomfort to severe secondary complications such as intestinal obstruction, perforation, or bleeding [4]. Preoperative diagnosis of gastrointestinal Burkitt lymphoma is challenging due to its mimicry of other diseases, often resulting in a diagnosis based on postoperative histopathological examination [5].

We report a 5-year-old male presenting with an ileocolic intussusception due to Burkitt lymphoma acting as the lead point.

This case report was prepared in accordance with the revised Surgical Case Report (SCARE) 2025 guidelines [6].

## 2 Case presentation

A 5-year-old male child from Ethiopia was presented to the hospital on January 2024 by his parents with a chief complaint of crampy abdominal pain and frequent vomiting of gastric contents of 24 h duration prior to hospital visit. In addition to these symptoms, he was unable to pass stool normally and had diarrhea containing currant jelly-like material and mucus. Examination in the emergency department revealed an irritable and lethargic child exhibiting colicky abdominal pain. On examination, the child was febrile (38 °C) with a blood pressure of 95/55 mmHg (using a pediatric cuff), tachypneic (respiratory rate of 28 breaths/min), and tachycardic (125 bpm) with normal oxygen saturation. Anthropometric measurements were within normal limits. The remainder of the physical examination was unremarkable except for abdominal distension with mildly tender on palpation. Initial laboratory investigations revealed a mildly elevated white blood cell count of 12,000 cells/μL, with normal hemoglobin and platelet levels. Blood group was O positive, and renal function tests, liver enzymes, random blood sugar, and serum electrolytes were within normal limits.

**Fig. 1:**
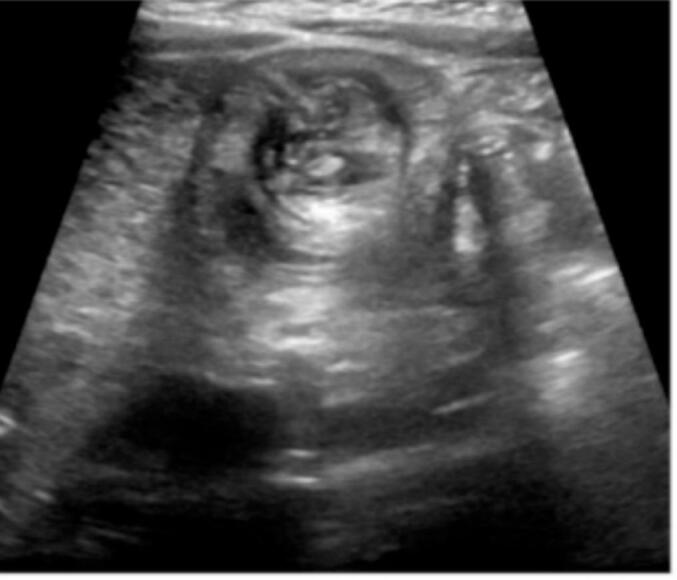
Abdominal ultrasound showed the classic “target sign” of intussusception at the ileocecal region.

**Fig.2:**
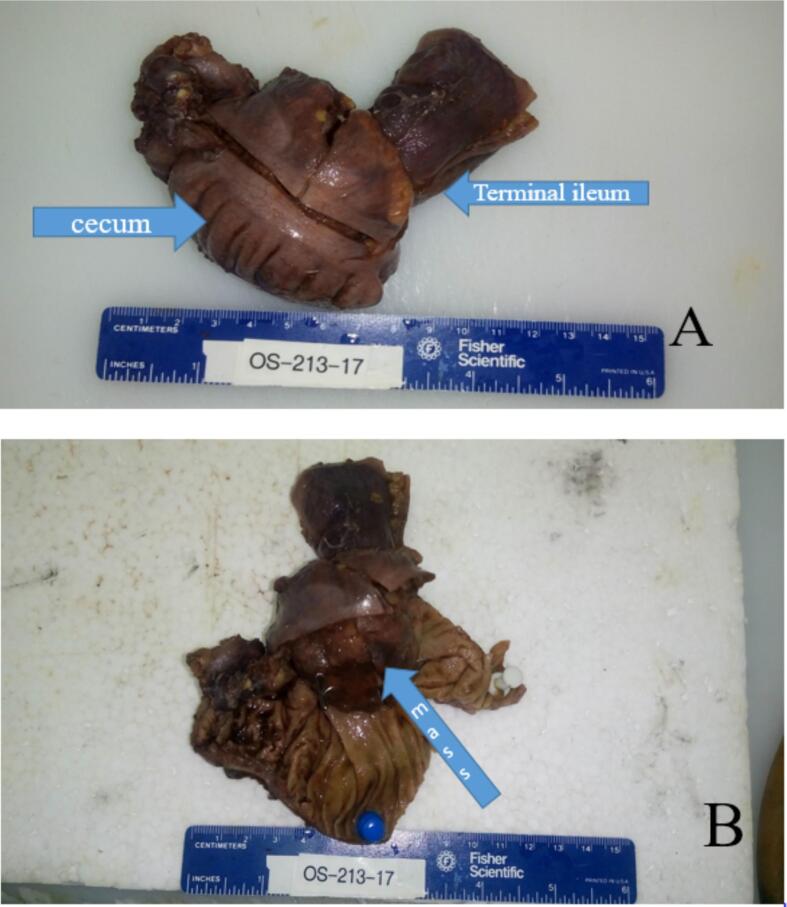
A. Shows gross surface of the resected segment consisting of terminal ileum and cecum. Appendix was unremarkable and not included in this photograph. B. Shows opened up surface of the mass at the ileocecal valve area which was non-reducible during grossing.

**Fig.3:**
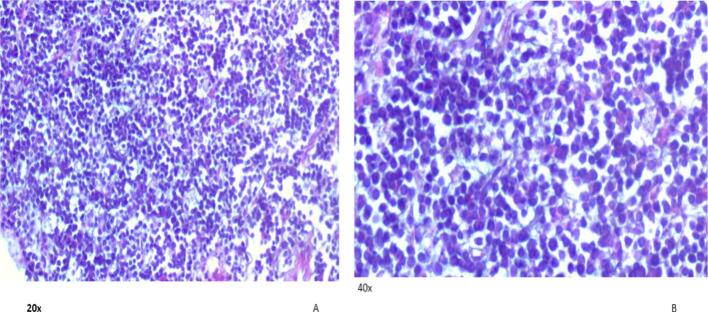
A. 20× magnification shows monotonous sheets of intermediate sized lymphoid cells. B.40× magnification shows tingible body macrophages some with cleared cytoplasm as artifactual clearing during tissue preparation giving starry sky pattern.

Following initial resuscitation, abdominal ultrasound revealed the classic signs of intussusception (Fig. 1). Given the absence of CT scan facilities, limited available expertise, and the patient's young age of 5 years, the likelihood of a secondary lead point was considered higher. Therefore, radiological reduction was not attempted. After administering the first dose of antibiotics, the patient was taken to the operating theater. Intraoperative findings confirmed ileocecal intussusception caused by an intraluminal mass located at the terminal ileum, which served as the lead point. A segmental resection of the ileocecal region with primary anastomosis was then performed. An attempt at manual reduction of the intussusception was unsuccessful; therefore, segmental resection of the affected bowel segment with primary anastomosis was performed. Upon opening the resected specimen, the mass was found at the terminal ileum, with involvement of the ileocecal valve, which was difficult to preserve. Histopathological examination of the resected specimen revealed the following: Macroscopic Findings: The specimen consisted of a segment of ileum, cecum, and appendix. The terminal ileum measured 5.2 × 3.5 cm, the cecum 9 × 4 cm, and the appendix 8 × 1 cm. The serosal surface was dark brown, and no mesentery was attached. A firm, gray-brown polypoid mass measuring 4 × 3 cm was identified at the ileocecal region, located 5 cm from the cecal resection margin and 4.5 cm from the ileal resection margin (Fig. 2A and B).Microscopic Findings: Microscopic sections showed a monotonous population of medium-sized lymphoma cells with an open chromatin pattern and frequent mitotic activity. Numerous scattered tangible body macrophages created a characteristic “starry sky” appearance (Fig. 3). After the diagnosis of Burkitt lymphoma, HIV and EBV serology tests were performed and both were negative. Immunohistochemistry was not conducted due to unavailability locally and the inability to afford sending the samples abroad for testing. Following the surgical procedure and confirmation of Burkitt lymphoma diagnosis, the patient was referred to a tertiary care center specializing in hematology and oncology. At this center, the patient received chemotherapy treatment and continued to be monitored and followed up by the specialized medical team. Postoperative follow-up at 1 week, 1 month, and every 3 months for 1 year revealed no complications, and the patient resumed normal activities.

## 3 Discussion

Burkitt lymphoma acting as a lead point for ileocecal intussusception is a rare secondary cause of intussusception in pediatric patients. Preoperative diagnosis is uncommon, and the diagnosis is usually made postoperatively after surgical treatment for intussusception. Consistent with this pattern, our patient presented with nonspecific acute abdominal symptoms and was diagnosed with Burkitt lymphoma after surgery. Burkitt lymphoma, a very aggressive form of B-cell non-Hodgkin lymphoma, was initially identified in 1958 by Dr. Dennis Burkitt while studying Ugandan children presenting with unusual jaw sarcomas [7]. Several risk factors are associated with Burkitt lymphoma, including Epstein-Barr virus (EBV) infection in immunocompromised individuals, while chronic inflammatory conditions like inflammatory bowel disease (IBD) can increase the risk of gastrointestinal lymphoma [8]. Unlike many cases of Burkitt lymphoma, our patient was immunocompetent and had no other identifiable predisposing factors for the disease. Burkitt lymphoma can manifest with a wide spectrum of clinical features, ranging from readily apparent peripheral lymphadenopathy accompanied by systemic symptoms (including fever, night sweats, and appetite loss) to more complex presentations involving organ dysfunction, such as renal impairment or central nervous system involvement due to metastasis. Rarely, Burkitt lymphoma can present atypically, as in our patient, with an intestinal polyp that predisposed to intussusception and acute abdomen secondary to intestinal obstruction [3]. The diagnosis of Burkitt lymphoma typically relies on histopathological and immunohistochemical analysis [5]. However, diagnosing gastrointestinal Burkitt lymphoma can be challenging, as illustrated by our patient, and is often made following laparotomy performed for acute intestinal obstruction, as our patient presented with ileocolic intussusception leading to obstruction [5]. Our case aligns with a subset of previously published reports describing primary gastrointestinal Burkitt lymphoma presenting with acute intestinal obstruction resulting from a pathological lead point. This presentation has been observed in a 14-year-old Tunisian child [9]. However, other cases detail a more insidious onset, with an 11-year-old Syrian child [10] and a 48-year-old adult [11] presenting with a protracted course of abdominal discomfort and a palpable mass. Despite these differing presentations, surgical intervention was the mainstay of treatment across all cases, and no recurrence was reported.

In the evaluation of intussusception, the choice of preoperative imaging depends on the clinical presentation [5]. While abdominal ultrasound is often the initial modality, cross-sectional imaging, ideally with CT scanning, is typically utilized to characterize any identified mass, assess the severity of the disease, and determine the stage of Burkitt lymphoma [5]. However, in our resource-constrained setting, immediate CT scanning was not feasible, and given the acute presentation of our patient, the decision was made to proceed directly to laparotomy for treatment of the intussusception.

Management strategies for gastrointestinal Burkitt lymphoma are tailored to the patient's presentation, stage of disease, and the presence or absence of complications [12]. Our patient, who presented with early-stage disease and intestinal obstruction necessitating surgical intervention, underwent complete resection of the presumed intestinal polyp [12]. This approach resulted in an excellent outcome, with no evidence of recurrence or metastasis and a sustained good quality of life.

## 4 Conclusion

In conclusion, Burkitt lymphoma is an aggressive B-cell non-Hodgkin lymphoma with diverse clinical presentations. Although rare, it can serve as a secondary lead point for intussusception, as seen in our patient. Proper postoperative evaluation in cases of secondary intussusception is crucial for accurate diagnosis. An appropriate histopathological diagnosis combined with complete surgical excision can result in an excellent prognosis. However, preoperative diagnosis of primary gastrointestinal Burkitt lymphoma remains challenging, especially in resource-limited settings, underscoring the need for improved diagnostic tools and approaches.

### 4.1 Strength and limitation

The strength of this case report lies in highlighting a rare clinical entity in a resource-limited setting, providing valuable insights for similar environments. The primary limitation of this report is the lack of immunohistochemical studies. Immunohistochemistry is an important diagnostic tool for confirming the diagnosis, determining the subtype, and informing optimal management strategies for this condition. Unfortunately, due to resource limitations in our setting and the patient's financial constraints, we were unable to pursue immunohistochemical analysis, including sending samples for evaluation abroad.

## Abbreviations

BL: Burkitt lymphoma
CBC: Complete Blood Count
EBV: Epstein-Barr virus
GI: Gastrointestine

## Consent

Written informed consent was obtained from the patient's family for publication and any accompanying images. A copy of the written consent is available for review by the Editor-in-Chief of this journal on request.

## Ethical approval

Ethical approval for this study was provided by the Debre Tabor university Department of Internal medicine Ethical review committee (DtU-IMR/2025), Debre Tabor university, Ethiopia, on June 20, 2025.

## Funding

There is no source of funding found for this paper.

## Author contribution

AAA: Conceptualization, design of the study, acquisition of data, drafting the article, revising it critically for important intellectual content, approval of the version to be submitted.

ZAE: Analysis, interpretation of data, drafting the article, revising it critically for important intellectual content, approval of the version to be submitted.

AMG: Conceptualization, analysis, drafting the article, revising it critically for important intellectual content, approval of the version to be submitted.

## Declaration of Generative AI and AI-assisted technologies in the writing process

AI language modelling tools were utilized for the improvement of English-language only in this case report.

## Guarantor

Addisu Assfaw Ayen, MD.

## Research registration number

N/A

## Conflict of interest statement

All authors declare that they have no conflict of interest.
